# Seroprevalence and prevalence of *Babesia vogeli* in clinically healthy dogs and their ticks in Costa Rica

**DOI:** 10.1186/s13071-021-04936-7

**Published:** 2021-09-14

**Authors:** Andrea García-Quesada, Ana Jiménez-Rocha, Juan José Romero-Zuñiga, Gaby Dolz

**Affiliations:** 1 Laboratorio de Entomología, Escuela de Medicina Veterinaria, Universidad Nacional, Heredia, Costa Rica; 2 Laboratorio de Parasitología, Escuela de Medicina Veterinaria, Universidad Nacional, Heredia, Costa Rica; 3 Programa en Medicina Poblacional, Universidad Nacional, Heredia, Costa Rica

**Keywords:** Tick-borne diseases, Serology, DNA, Piroplasmida

## Abstract

Canine babesiosis is a disease caused by a parasite of the genus *Babesia* which destroys red blood cells. Previous studies have shown the presence of *Babesia vogeli* in rural areas in Costa Rica using molecular techniques. The objective of the present study was to determine the seroprevalence and prevalence of *B. vogeli* in clinically healthy dogs and their ticks at the national level, both within and outside the Central Valley. Blood samples and ticks from 482 dogs were collected between June 2011 and May 2014, and analyzed by immunofluorescence assay (IFA) and real-time polymerase chain reaction (qPCR); two protocols of endpoint PCR and sequencing were used to confirm qPCR-positive samples. Seroprevalence of canine babesiosis of 5.3% (24/453) was determined at the national level, specifically 2.0% (5/253) within and 9.5% (19/200) outside the Central Valley, respectively. Real-time PCR determined a global prevalence of *B. vogeli* of 31.3% (125/400): 21.4% (47/220) within the Central Valley and 43.3% (78/180) outside the Central Valley. The endpoint PCR amplified only 10 of the 125 blood samples identified as positive in qPCR. One sample amplified by endpoint PCR was sequenced and identified as *B. vogeli*. Twelve canines were identified with past infections, seven canines with active infection, and 111 canines with early infection. Two species of ticks were found with *B. vogeli*: *Rhipicephalus sanguineus* sensu lato (*n* = 40) and *Amblyomma ovale* (*n* = 1). The prevalence of canine babesiosis at the national level, both within and outside the Central Valley, is reported here for the first time, determining the presence of the piroplasmid throughout the country, with a higher circulation of the agent outside the Central Valley. Only one species, *B. vogeli*, was detected in the blood of dogs and their ticks. Therefore, veterinarians should consider using qPCR to determine the presence of the parasite in blood donors and before starting treatment of vector-borne disease in dogs.

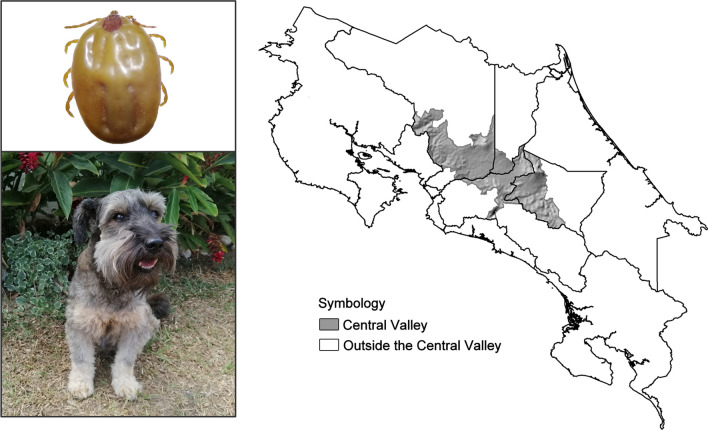

Babesiosis is caused by intracellular protozoa of the genus *Babesia*, which infect and destroy the infected red blood cells of susceptible hosts. *Babesia vogeli*, a cosmopolitan species transmitted by the tick *Rhipicephalus sanguineus* sensu lato, is the cause of babesiosis in dogs [1, 2].

In Latin America, the prevalence of *B. vogeli* has been reported recently in Argentina (7%) [3], México (10%) [4], Peru (1.4%) [5], Colombia (26%) [6], Brazil (16.7%) [7], Paraguay (5.5%) [8], and Chile (6.3%) [9] through molecular techniques. In Central America, *B. vogeli* was detected using molecular techniques in Nicaragua (15.3%, 6/39) [10] and in Costa Rica (20.0%, 8/40) [11]. Another study using molecular techniques established a prevalence of 8.2% (12/146) of *B. vogeli* in dogs in four Costa Rican cities. However, it was not possible to establish this pathogen in the Central Valley of Costa Rica [12]. In addition, a recent study reported *B. vogeli* seropositivity of dogs presented randomly at selected veterinary clinics [13]. However, the authors did not provide data about *B. vogeli*-infected dogs and ticks. Furthermore, information about *B. vogeli* prevalence in clinically healthy dogs inside and outside the Central Valley is absent. Therefore, we investigated the *B. vogeli* prevalence in dogs and their ticks at the national level, both inside and outside the Central Valley.

The Central Valley, located in the center of the country and surrounded by several mountains and volcanos, is the largest developed area of Costa Rica, housing almost three-quarters of the Costa Rican population. It occupies 3237 km^2^ (equivalent to 6.3% of the national territory). The most important economic activities of the country take place in this area. It is also the seat of the most important cities and home to government offices and major institutions [14]. It is located between 900 and 1200 m above sea level, with temperatures ranging from 17 to 22 °C.

In contrast, regions outside the Central Valley are inhabited by a smaller and relatively poor population (30.3% poverty). These areas occupy most of the countryside. The altitude in these regions is between 0 and 900 m above sea level, and temperatures range between 20 and 27.5 °C [15].

A cross-sectional, observational, descriptive study was carried out with blood samples from dogs and their ticks collected between June 2011 and May 2014, in the framework of a project directed to detect causative agents of vector-borne diseases in dogs. In order to reach representativeness inside and outside the Central Valley, the sample size was estimated to be 386 individuals (50% expected prevalence, 95% confidence, 5% accepted error) over a calculated population of more than 40,000 dogs using Win Episcope 2.0. The number of dogs to be analyzed was proportionally allocated based on the proportion of households reported inside (62%) and outside (38%) the Central Valley and the estimated number of dogs (1.6) found per household [16]. Blood samples from 482 dogs were collected, stored at 4 °C until completion of the serum separation, and frozen at −20 °C until analysis. For the molecular and serological tests, 400 whole blood samples (220 within and 180 outside the Central Valley) and 453 serum samples (253 within and 200 outside the Central Valley) were analyzed. Both samples (blood and serum) were obtained from 371 randomly selected animals: 199 inside and 172 outside the Central Valley.

Each dog was examined for 10 min to collect the ticks, which were stored in collection tubes with 70% ethanol, and assigned the same code as the animal. Ticks were not allowed to digest the host's blood before analysis. A total of 623 ticks were found on the examined dogs. Further details of the study population, sampling methodology, and taxonomic tick identification are described in a previous study [16].

A total of 136 (28.2%) out of 482 dogs were found infested with ticks, 129 dogs with *R. sanguineus* s.l., four with *Amblyomma ovale*, one with *Ixodes boliviensis*, and two dogs with mixed infestations (one with *R. sanguineus* s.l. plus *A. ovale*, and the other with *R. sanguineus* s.l. plus *Amblyomma mixtum*). Ticks from each dog were separated by species, sex, and stage in 1.5 ml tubes. They were stored in the laboratory at −20 °C until DNA extraction and PCR were performed. When ticks of the same species but different sexes or stages were found on dogs, only one group of ticks was analyzed by PCR according to the following priority: females > nymphs > males > larvae.

Indirect immunofluorescence assay (IFA, MegaScreen^®^ Fluo BABESIA canis, and MegaScreen^®^ Fluo BABESIA vogeli; Megacor Diagnostik, Horbanz, Austria) was used for the detection of antibodies in dog serum, following the manufacturer's instructions. All sera were initially evaluated in a 1:32 dilution in phosphate-buffered saline (PBS), pH 7.4. Sera that showed reactivity were evaluated in serial twofold dilutions from 1:32 to 1:4096. A non-reactive dog serum (negative control) and a reactive dog serum (positive control) were included at a 1:32 dilution in each slide. Seroreactive samples were defined as having endpoint titers ≥ 1:32, based upon positive thresholds defined by the manufacturer.

DNA was extracted from blood samples and ticks using the DNeasy^®^ Blood and Tissue Kit (Qiagen, Chatsworth, CA, USA), following the manufacturer's recommendations. The quantity and quality of DNA of all blood and tick samples were verified by measuring absorbance at 260 nm and stored at −20 °C until polymerase chain reaction (PCR) analyses.

DNA samples were analyzed by real-time PCR (qPCR), and positive qPCR samples confirmed by two endpoint PCR protocols. The qPCR amplified a region of the rRNA gene of *B. vogeli* of approximately 102 bp [17]. The reaction was carried out in a final volume of 20 μl, adding 12 µl of Maxima SYBR Green/ROX qPCR Master Mix (2×) (Thermo Scientific), 1 µl of each primer (10 uM), 5 µl of the extracted DNA (40 ng/µl), and 1 µl of nuclease-free water (Thermo Scientific). *Babesia vogeli* DNA-positive control donated by Dr. Gad Baneth from the Hebrew University of Jerusalem was used as a positive control, and nuclease-free water (Thermo Scientific, USA) as no-template control. A standard curve was elaborated to determine the efficiency of the qPCR, and the specificity of the technique was then assessed with a dissociation curve analysis. The qPCR showed efficiency of 103%. Additionally, the dissociation curves showed only one amplified product in the samples with the DNA of interest. Samples with threshold cycle (Ct) values between 15 and 33 were considered positive.

Positive samples with qPCR were subjected to two endpoint PCR protocols. One protocol of the endpoint PCR [18] to amplify a segment of 450 bp of the 18S rRNA gene of *Babesia* spp. was used. A second PCR protocol was used to amplify a 1600 bp fragment of the 18S rRNA gene [19]. Amplified sequences of both PCRs were sent to Macrogen Inc. (Seoul, South Korea) to be purified and sequenced.

Sequences were edited using the BioEdit Sequence Alignment Editor^®^ program [20], and were compared with the National Center for Biotechnology Information (NCBI) database using the BLASTn algorithm. The alignment was made using the MUSCLE program [21]. Finally, a sequence obtained and edited was deposited in GenBank.

Descriptive statistics were performed by calculating central tendency (average) and dispersion (standard deviation) measures. The relative frequency of molecular and serological prevalence and proportions of ticks with *Babesia* were determined, with their respective 95% confidence intervals. In addition, the association of dogs living outside the Central Valley with the agent was estimated using an unconditional binomial logistic regression model. Results with *P* < 0.05 were considered to be statistically significant in all tests. All analyses were performed in Infostat software [22].

*Babesia* spp. seroreactivity was detected in 5.3% (24/453) of dogs nationwide. A lower seroprevalence was found in dogs living in the Central Valley (2.0%, 5/253) in comparison with dogs living outside the Central Valley (9.5%, 19/200) (*P* < 0.001).

The province that showed the highest seroprevalence was Guanacaste (16.9%, 13/77), followed by Puntarenas (9.8%, 6/61), Heredia (3.3%, 1/30), Alajuela (2.6%, 1/38), and San José (1.7%, 3/172); no seropositive dogs were found in Cartago (0/29) or Limón (0/46). Eighteen (75.0%) of the positive sera showed high titers (1:128 to 1:4069); of these, four (22.0%) belonged to dogs living in the Central Valley, and 14 (78.0%) outside the Central Valley. Dogs living outside the Central Valley showed a strong association with seropositivity (OR = 5.2; 95% CI 1.9–14.2).

*Babesia vogeli* DNA was detected in 31.2% (125/400) of dogs nationwide with qPCR, establishing a prevalence of 21.0% (47/220) and 43.0% (78/180) inside and outside the Central Valley, respectively. The province that showed the highest prevalence was Alajuela (56.3%, 18/32), followed by Cartago (52.4%, 11/21), Puntarenas (45.0%, 25/56), Guanacaste (39.0%, 27/69), Limón (38.0%, 16/42), and San José (16.0%, 24/153), and the lowest prevalence was detected in Heredia (15.0%, 4/27). Even as in the serological determination, dogs living outside the Central Valley were more likely to be qPCR-positive (OR = 2.81; 95% CI 1.82–4.36).

Only 10 and one of the 125 samples identified as positive in qPCR (Ct values of 15 to ≤ 33) were determined to be positive in the 18S rRNA PCR (450 bp) and 18S rRNA PCR (1600 bp), respectively. One positive sample in the 18S rRNA PCR (450 bp) was sequenced (GenBank: MT785903) and identified as *B. vogeli* (100% sequence identity and 100% query cover) when compared with those (e.g., GenBank: MK495837.1 and LC331058.1) deposited in GenBank.

Out of 138 tick samples, 40 identified as *R. sanguineus* s.l. and one as *A. ovale* were detected as positive by qPCR. Of these, 34.0% (16/47) were found with *B. vogeli* inside the Central Valley and 27.5% (25/91) outside the Central Valley.

Seven ticks yielded positive results in the 18S rRNA PCR (450 bp), but none of the DNA sequences was suitable for sequencing. No ticks were detected as positive by the 18S rRNA PCR (1600 bp).

Twelve dogs were seropositive and qPCR-negative. From these, only one dog had positive ticks. Most seropositive and qPCR-negative dogs were detected outside the Costa Rican Central Valley (9/12). Seven canines were found seropositive and qPCR-positive for *B. vogeli*. Two of them had qPCR-positive ticks, and 86.0% (6/7) were detected outside the Central Valley. One hundred eleven dogs were qPCR-positive and seronegative, of which 62.2% (*n* = 69) were detected outside the Central Valley, and 10% (*n* = 11) had qPCR-positive *R. sanguineus* s.l. ticks.

Before the present study, the prevalence of *B. vogeli* in healthy dogs had not been determined in Costa Rica, either in the total population or inside and outside the Central Valley. The seroprevalence determined at the national level (5.3%) was lower than the seropositivity reported recently by Springer et al. [13], probably due to the different dog populations analyzed in the two studies. The majority of dog samples analyzed came from rural areas. Most seropositive dogs were found in the provinces of Guanacaste (16.9%, 13/77) and Puntarenas (9.8%, 6/61), which agrees with Springer et al. [13]. Seropositive dogs were five times as likely to live outside the Central Valley as seronegative dogs. These areas present less favorable socioeconomic conditions than areas in the interior of the Central Valley. Generally, people in rural areas do not supervise their dogs closely, providing food, shelter, and preventive medicine less often than urban dwellers [23]. In addition, climatic conditions occurring outside the Central Valley (high humidity and temperature) may favor a high level of tick infestation of animals [24]. In contrast, temperatures lower than 18 °C, which occur in the Central Valley, prevent the normal biological cycle of *R. sanguineus* s.l. [25].

The prevalence of *B. vogeli* (31.2%, 125/400) found through qPCR at the national level was higher than that reported in previous studies. These differences could be due to the size of the samples analyzed [11], the representability by regions [12], or the molecular technique used [13]. It is also possible that when the samples were taken in 2011, the infection was beginning to spread; at that point, many dogs were positive in qPCR and seronegative, which agreed with the higher seroprevalence determined in 2014 in dogs from Costa Rica [13].

A higher prevalence of *B. vogeli* was determined outside the Central Valley than that found within the Central Valley (*P* ≤ 0.05). qPCR-positive canines were found to be three times as likely to live outside the Central Valley as qPCR-negative canines. This study represents the first to report *B. vogeli* in dogs from the Central Valley.

Until now, *B. vogeli* had not been reported in ticks of canines in Costa Rica. However, the DNA of *B. vogeli* was found in 29.7% of the ticks, mainly *R. sanguineus* s.l. ticks (97.6%), which are considered natural vectors of the parasite [26].

Only 8.0% (10/125) of qPCR-positive dogs and 17.1% (7/41) of qPCR-positive tick samples were confirmed by endpoint PCR. The remaining samples were not detected as positive by endpoint PCR. These results could be due to the higher sensitivity of qPCR over endpoint PCR [27, 28]. Therefore, it is recommended that this technique be used for routine diagnosis in veterinary practice.

When comparing the IFA results with the qPCR, a much higher prevalence (31.2%) was established than for seroprevalence (5.3%). These findings could indicate that many dogs (111) were detected in an early infection phase, suggesting a recent subclinical spread of the agent in the population at the time of sample collection. This agrees with the findings of qPCR-positive ticks on seronegative and qPCR-negative canines. The literature reports that ticks need to feed at least 3 days on their canine hosts to transmit *Babesia*, whereas antibodies develop 2 to 3 weeks after infection [29]. A total of seven canines were both seropositive and qPCR-positive, indicating an active infection. The animals are thought to have probably been infected at least 21 days earlier, which is how long it takes for anti-*Babesia* spp. antibodies to appear in the dog, whereas past infections were established in 12 dogs (seropositive and qPCR-negative) [30].

The results obtained in this study with a healthy dog population show that *B. vogeli* infections have increased in Costa Rica in recent years. This went relatively unnoticed by veterinarians, probably due to the subclinical presentation of the infection from 2011 to 2014. However, it is crucial to consider this protozoan in the differential diagnosis of vector-borne diseases, since they can cause disease in young, adult, or immunocompromised dogs if they receive *B. vogeli*-positive blood transfusions, because the specific treatment is different from rickettsial agents. The use of qPCR is an accurate diagnostic tool for the detection of this piroplasmid. Considering that approximately 25% of the Costa Rican territory corresponds to protected wild areas, these results also indicate a risk of infection of our wild canids. Therefore, we recommend conducting future studies in those populations [2].

## Data Availability

The 18S rRNA sequence of *B. canis vogeli* obtained in this study was deposited in GenBank (accession number MT785903).

## Abbreviations

IFA: Indirect immunofluorescence assay
PCR: Polymerase chain reaction
qPCR: Real-time PCR
Ct: Threshold cycle
NCBI: National Center for Biotechnology Information
18S: Small subunit ribosomal
rRNA: Ribosomal ribonucleic acid
